# Computer assisted navigation in total knee and hip arthroplasty

**DOI:** 10.1051/sicotj/2017034

**Published:** 2017-07-28

**Authors:** Kamal Deep, Shivakumar Shankar, Ashish Mahendra

**Affiliations:** 1 Golden Jubilee National Hospital Agmemnon Street, Clydebank G81 4DY Glasgow UK; 2 Glasgow Royal Infirmary 84 Castle Street G4 0SF Glasgow UK

**Keywords:** Computer assisted surgery, Navigation, Knee arthroplasty, Hip arthroplasty, Kinematics, Robotics

## Abstract

*Introduction*: Computer assisted surgery was pioneered in early 1990s. The first computer assisted surgery (CAS) total knee replacement with an imageless system was carried out in 1997. In the past 25 years, CAS has progressed from experimental in vitro studies to established in vivo surgical procedures.

*Methods*: A comprehensive body of evidence establishing the advantages of computer assisted surgery in knee and hip arthroplasty is available. Established benefits have been demonstrated including its role as an excellent research tool. Its advantages include dynamic pre-operative and per-operative assessment, increased accuracy in correction of deformities, kinematics and mechanical axis, a better alignment of components, better survival rates of prostheses and a better functional outcome. Adoption of computer navigation in the hip arthroplasty is still at an early stage compared to knee arthroplasty, though the results are well documented. Evidence suggests improved accuracy in acetabular orientation, positioning, hip offset and leg length correction.

*Results*: Among the orthopaedic surgeons, navigated knee arthroplasty is gaining popularity though slowly. The uptake rates vary from country to country. The Australian joint registry data shows increased navigated knee arthroplasty from 2.4% in 2003 to 28.6% in 2015 and decreased revision rates with navigated knee arthroplasty in comparison with traditional instrumented knee arthroplasty in patient cohort under the age of 55 years.

*Conclusion*: Any new technology has a learning curve and with practice the navigation assisted knee and hip arthroplasty becomes easy. We have actively followed the evidence of CAS in orthopaedics and have successfully adopted it in our routine practice over the last decades. Despite the cautious inertia of orthopaedic surgeons to embrace CAS more readily; we are certain that computer technology has a pivotal role in lower limb arthroplasty. It will evolve to become a standard practice in the future in various forms like navigation or robotics.

## Introduction

The prevalence of osteoarthritis (OA) in both North America and Europe is predicted to increase approximately 40% from 2005 to 2030 [1]. Total knee arthroplasty (TKA) and total hip arthroplasty (THA) remain the preferred treatment for end-stage knee and hip OA after failed non-surgical treatment. Joint replacement surgery reliably relieves pain, aids patients to return to near normal function and improves the health-related quality of life [2, 3]. THA is very successful and is associated with reproducible clinical outcomes with over 95% survivorship at 10-year follow-up and 80% survivorship at 25-year follow-up [2, 3].

An estimated increase in primary THA by 200% and TKA by as much as 673% is predicted from 2005 to 2030. Revision THA and TKA are projected to increase by 137% and 601%, respectively, between 2005 and 2030 [4]. Currently in the UK, 35% of patients under the age of 65 years are having hip and knee replacements and 12% of those are below the age of 55 years. There is very high probability of these patients requiring revision surgery in future [5]. The younger patient groups, especially males below the age of 55 years, have a 10-year survivorship and 16-year survivorship of 80% and 33%, respectively [6]. The patient expectations vary between higher demand younger patients and elderly patients. High expectations from TKA and THA pose a tough challenge to deliver functionally. TKA failure is multifactorial but more than 50% of early revisions are secondary to instability, malalignment or malposition, and failure of fixation [7], most of which result from poor surgical technique. THA failure is multifactorial. Early revisions at less than five years are due to implant malposition, dislocation and infection [8] which are dependent on the surgical technique. Good surgical technique and accurate implantation of the components are likely to improve the longevity and function of both TKA and THA. With such a huge revision rate predicted, it is imperative for orthopaedic surgeons to get it right the first time. Computer navigation provides evidence-based advantages to help surgeons achieve this goal.

Computer assisted surgery (CAS) has been around since early 1990s. The first *in vitro* CAS was performed in 1991 by Professor Nolte at Muller’s laboratory with the insertion of computer navigated pedicle screw into a saw bone vertebra [9]. This marked the beginning of a new era of innovation in the field of CAS in orthopaedics. Numerous novel ideas, refinement of processes and equipment led to the first image-free system for total knee replacement in 1997 by Picard and Saragaglia, who pioneered this technology that demonstrated promising future prospects [10]. Twenty years on, the technological advances in computer assisted orthopaedic surgery (CAOS) have consistently produced excellent outcomes in TKA, THA, knee osteotomies, spinal surgery and oncological surgery. New applications are being explored in anterior cruciate ligament reconstruction, ankle, shoulder and trauma surgery. We explore the role and evidence of CAOS in TKA and THA.

## Material and methods

The objective criteria for surgeon dependent factors influencing TKA and THA are broken down into individual entities. These lead to reproducible improvement of the mechanical, clinical and functional outcome.

With TKA the surgeon dependent factors influencing outcome are divided into pre-operative, per-operative and post-operative factors.

1. Pre-operative assessment of patient’s anatomy, deformity (static and dynamic) and function using clinical, radiological and objective function assessments. The dynamic deformity can be assessed with navigation using extra-cutaneous trackers in clinic or gait lab.
2. Per-operative assessment and near normal reconstruction of patient’s anatomy, alignment, ligament balance, mechanics and kinematics.
3. Post-operative clinical, radiological and functional outcome assessment.

The surgeon dependent factors in THA for optimal clinical, radiological and functional outcome are:

1. Acetabular implant orientation and positioning of the centre in the intended place.
2. Restoration of the patient’s hip offset and optimal leg length.

### TKA

#### Pre-operative accurate static and dynamic detection of patient’s anatomy and deformity

Most of the existing conventional pre-operative methods of assessment are static which include radiographs (antero-posterior knee view, lateral view, skyline patella femoral joint view and long leg alignment view). The reliability of these radiographs in pre-operative planning is controversial [11, 12].

The patient specific instrumentation (PSI) has been described in the literature. The PSI technology requires pre-operative computerised tomography (CT) or magnetic resonance imaging (MRI) scan to enable the manufacturing of patient specific cutting jigs. PSI adds increased pressure on the radiology department, increases cost and, for the patient, requires additional hospital visits for the scan in addition to the radiation exposure of the CT scan. In some studies, PSI has produced less accurate correction of alignment of TKA components in comparison with traditional instrumentation [13]. The role of PSI in TKA is still unclear in the literature.

Computer navigation offers pre-operative dynamic assessment of deformity, alignment and kinematics. A study on normal individuals enabled dynamic assessment of knee laxity and femoro-tibial mechanical axis (FTMA) at 0° extension and 15° of knee flexion [14]. This has been described with a standardised technique using fibroelastic straps around femur and tibia stabilising extra-cutaneous markers that allow 6° of freedom of movement and a device producing 10 Nm torque to assess knee ligament laxity [14]. The potential role of navigation technology in the dynamic assessment of osteoarthritic knees in the outpatient clinic setting offers exciting opportunity in future to be able to set target values to reproduce during the surgery.

#### Per-operative assessment, guidance of bone cuts and near normal reconstruction of patient’s anatomy, alignment, ligament balance and mechanics

CAOS aids intra-operative dynamic accurate assessment of anatomy of individual patients differentiating subtle variations between various patients. Utilising standard anatomical landmarks, the computer designs a three-dimensional (3D) image of the anatomy [15]. CAOS provides dynamic and real-time assessment of femoral-tibial mechanical axis (FTMA), measurements of coronal and sagittal knee alignment offering a potential alternative to radiographs [16], tibial rotational profile [17] and knee kinematics [18].

A pre-implantation assessment of knee coronal plane deformity through the range of movement has been analysed and classified to assess kinematics [18]. This *Deep’s* classification describes four main types (1, 2, 3 and 4) with eight sub-groups (1A, 1B, 2A, 2B, 3, 4A, 4B and 4C); Table 1 [18].

**Table 1.** Deep’s classification of knee deformity as it flexes from an extended position.

Main group	Class/Type	Coronal deformity as the knee flexes from extension to 90° flexion
Neutral		
Varus/Valgus		
1	1A	Deformity remains same
	1B	Deformity increases
2	2A	Deformity decreases but does not reach neutral
	2B	Deformity decreases and reaches neutral
3	3	Deformity becomes opposite deformity (Varus becomes Valgus and Valgus becomes Varus)
4	4A	Deformity 1st increases then decreases but does not reach neutral
	4B	Deformity 1st increases and then decreases to reach neutral
	4C	Deformity 1st increases and then becomes opposite deformity (Varus becomes Valgus and Valgus becomes Varus)

**Figure 1.**
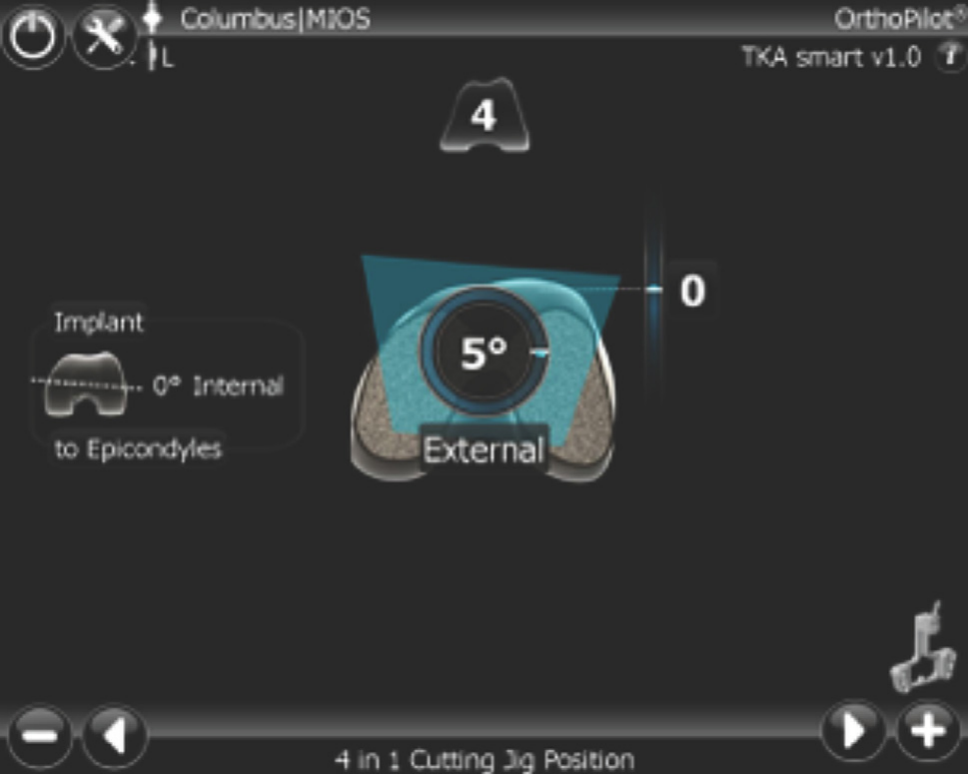
The implant size and positioning of the rotational position of femoral cutting jig.

CAOS works with both gap balanced resection or measured bone resection. Live information of resection gaps, implant size and three-dimensional alignment of implants can be seen on a computer screen and allows bespoke changes for each patient (Figure 1) [15].

**Figure 2.**
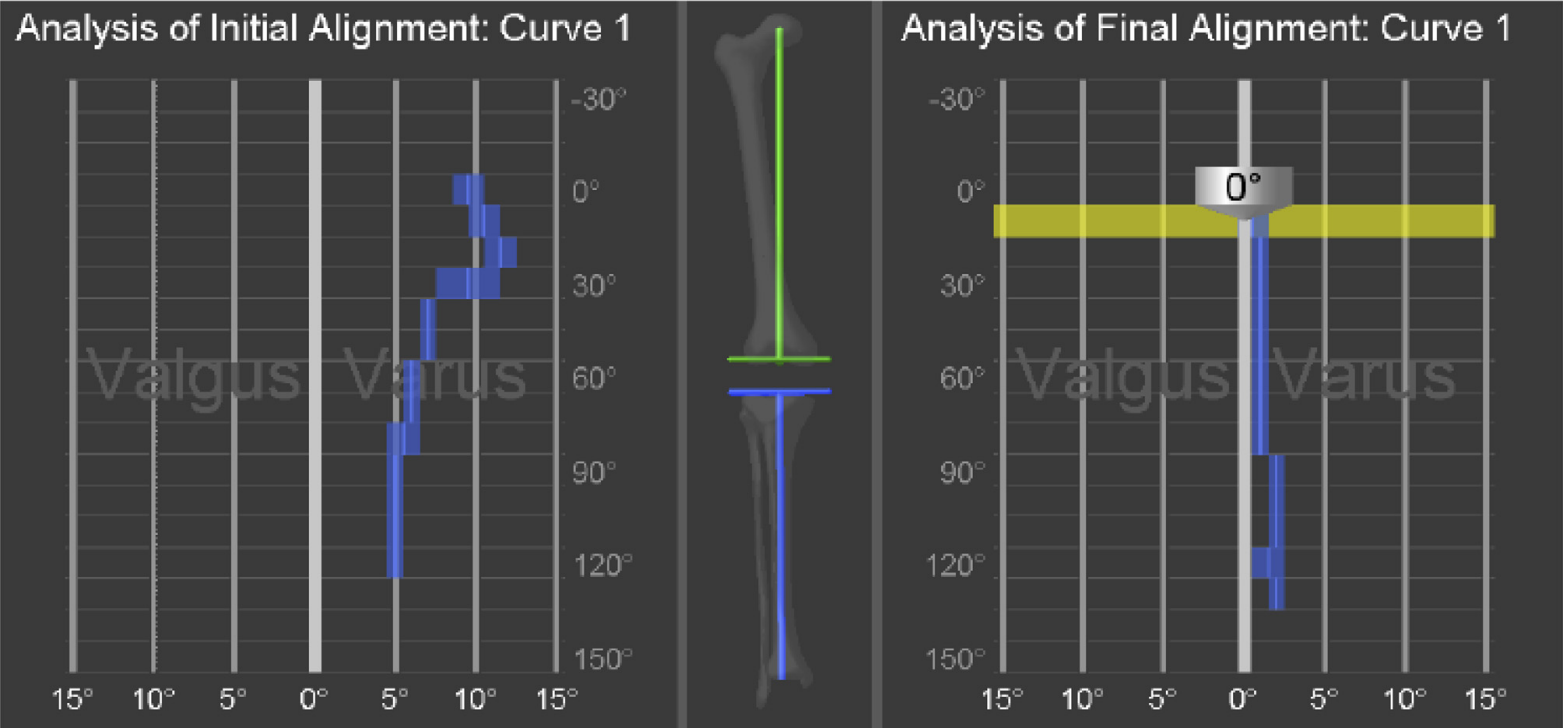
The change in coronal deformity (*X* axis) of knee femoro-tibial mechanical alignment angle, as the knee flexes (*Y* axis): first part showing before the surgery valgus deformity first increases and then decreases as the knee flexes (Type 4A in Deep’s classification) and second part showing the axis achieved after surgery of same patient (Neutral alignment).

Ligament release and soft tissue balancing are traditionally done based on deformity in extension and 90° flexion. Extensive collateral ligament releases are associated with post-operative haematoma, increased risk of infection and wound complications [19]. Extensive soft tissue release may increase hospital stay and may necessitate more constrained TKA implants. The dynamic change during flexion influences collateral releases. The dynamic assessment in CAOS achieves coronal deformity correction and soft tissue balance without needing extensive collateral soft tissue release [20]. Navigation allows post-implantation assessment of restoration of FTMA and kinematics (Figure 2) [18].

#### Post-operative clinical, radiological and functional outcome assessment

**Figure 3.**
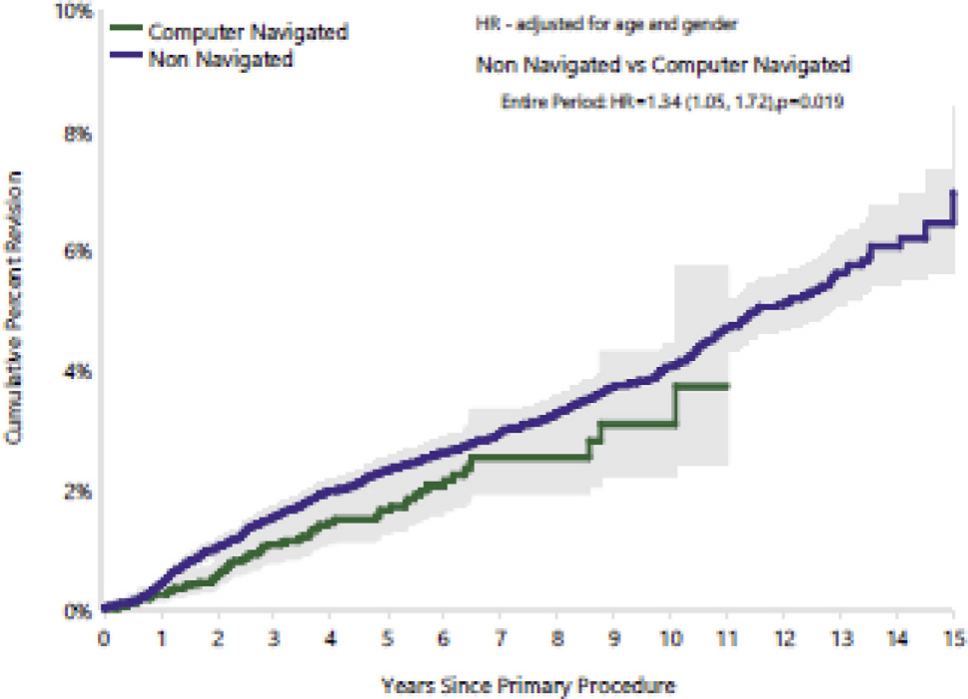
Australian Joint registry report 2016.

CAOS has proven post-operative clinical and radiological improvement of alignment of components [21, 22]. Decreased post-operative embolism [23] and blood loss were noted in all patients including ones with high Body Mass Index (BMI) [24, 25]. In the Australian joint registry, at 11 years post navigation TKA, the revision rates are lower than TKA done with traditional instrumentation in patients under 55 years of age [26] (Figure 3).

Earlier lack of evidence on post-operative functional improvement has been one of the reasons CAOS did not gain so much popularity. Some studies found no difference in functional outcome between CAOS group and traditional group [27, 28]. Outcome assessment factors are gross and not powered enough to detect the statistical difference in most small studies. These studies are underpowered for detection of functional improvement. But now there is evidence in the literature including meta-analysis that CAOS along with improved anatomical accuracy leads to functional improvement too [21, 29, 30].

### THA

#### Acetabular implant orientation and positioning of the centre in the intended place

Lewinnek et al. described the safe zone of acetabular cup insertion as anteversion 15° ± 10°; inclination 40° ± 10° [31]. Acetabular cup orientation is crucial to good performance and longevity of THA. Acetabular cup malposition outside of the Lewinnek’s safe zone increases the risk of complications like decreased range of movements, increased rates of impingement, polyethylene wear, acetabular migration, recurrent dislocations, pelvic osteolysis and early failure rates [32–34]. Acetabular cup orientation based on surgeon’s visual assessment often results in an inaccurate placement [35]. The natural acetabular orientation in arthritic hips is quite variable and knowledge of this using navigation provides a better understanding of this orientation in individual patients [36], which offers an advantage for precise positioning of the acetabular component. Several studies have demonstrated convincingly that the acetabular orientation is more precise with navigated THA [37, 38], acetabular component was beyond the Lewinnek’s safe zone only in 8.63% of hips in the navigated group, compared with 28.4% in the conventional group [39].

#### Hip offset and leg length correction

Traditionally offset in THA has always been described in relation to femoral offset. Hip offset is a concept which factors in acetabular offset and hip centre of rotation (COR). A leg length/offset discrepancy of more than 5 mm correlates with non-physiological kinematics of gait [40]. Other detrimental outcomes include low back pain, nerve injury, patient dissatisfaction and increased litigations [41]. Navigated THA achieved 95.39% hip offset within 6 mm and 96.04% leg length restored within 6 mm of control contra-lateral side [42]. Navigated THA reduces malpositioning and facilitates the insertion of components in near normal orientation in comparison with the traditional THA [39, 42]. Patients are usually on lateral position and the most accepted landmarks are anterior superior iliac spines (ASIS) and pubic symphysis in anterior pelvic plane [31]. In obese patients, difficult palpation of these landmarks is an issue [43]. In several other studies, difficulty in registering anterior pelvic plane in obese patients is not an issue [44, 45].

CAOS offers excellent teaching and training opportunities with resultant reduction of learning curve associated with improvement in cognitive skills. The real-time feedback improves precision and accuracy in surgical techniques. It is an excellent teaching and training tool [46]. There is a role for simulation surgical training to accelerate the learning curve and to reduce complications.

## Results

In summary, despite initial learning curve and increased operating time in initial surgeries, regular use of CAOS in TKA demonstrates increased accuracy of implant alignment leading to better knee function and improved longevity of implant in younger patients [26, 47, 48]. CAOS in THA aids precise acetabular cup placement with decrease in the number of outliers, better hip offset and leg length discrepancy [39, 42]. It leads to consistent reproducible results in the hands of both experienced navigated surgeon and trainee surgeon [49].

## Discussion

The probability of finding new medical devices approved 10 years ago is less than devices approved five years ago. Fifty percent of devices approved for introduction to the orthopaedic market place are unavailable at 10 years. Only 2% of them were deemed to have safety problems [50]. CAOS has been embraced cautiously by orthopaedic surgeons across the world. There is about 30% usage in Germany [51], 28.6% usage in Australia [26] and sporadic usage in the UK, North America, Brazil, France and Asia [51]. The common arguments for delayed adoption of CAOS are increased operative duration, risk of a fracture at tracker pin sites, cost and in the past no difference in functional outcome. There is a learning curve, after 20 cases a novice navigation surgeon and experienced navigation assisted surgeon had similar operative times; however, all the surgical implantations were precise even during the learning curve [52]. In any surgery irrespective of navigation the initial few cases take longer time, which is the case for conventional joint replacements as well. Somehow the expectation from navigation technology has been that it should be equal or take even less time than conventional from the very start, even in the initial cases. Some experienced orthopaedic surgeons unlike their younger counterparts, less acquainted with computer games and technology, may not like the idea of computers during surgery and thus resist CAOS. A risk of fractures at tracker pin sites is reported, but the risk was as low as 0.13% (one in 777) in one study. The fracture occurred in an 82-year-old female patient subsequent to a direct fall onto the operated knee requiring an intramedullary nail fixation [53]. This problem has been substantially reduced by use of bicortical tracker attachment pins. In our institution with over 5000 navigated surgeries, no case of fracture was noted. The initial setup cost incurred for navigation system proves to be advantageous in long term. The number of operating instrumentation trays can be reduced with navigation. Numerous recent studies have described improved functional outcome in the CAOS group compared to the traditional instrumented group [29, 30, 47]. The revision burden from failed TKA and THA includes costlier implants, multiple prolonged hospital admissions, antibiotics, blood products, social services and costs. Any technique to help reduce this should be helpful. Now the navigation technology has been integrated into the robotic surgery. The available orthopaedic robots all use navigation technology to guide cut orientation and to detect movements during surgery. Only add the cutting end as well to make it robotic. We believe the evidence of CAOS technology in orthopaedics is well established over two decades and our successful adoption in routine practice confirms the role of computer navigation in lower limb arthroplasty. This will evolve to become a standard practice in the future in various forms like PSI, 3D printing, accelerometer-based navigation, infrared-based application or robotics.

## Conflict of interest

Kamal Deep is a consultant for BBraun. He is the secretary of the CAOS International Society. The institution received research funding from Stryker, BBraun, Bayer, Mathys, Zimmer, Convatec, and BBT.
